# Lepidopterans as Potential Agents for the Biological Control of the Invasive Plant, *Miconia calvescens*

**DOI:** 10.1673/031.012.6301

**Published:** 2012-05-20

**Authors:** Elisangela G.F. Morais, Marcelo C. Picanço, Altair A. Semeão, Robert W. Barreto, Jander F. Rosado, Julio C. Martins

**Affiliations:** ^1^ Embrapa Roraima - Brazilian Agricultural Research Corporation's, Boa Vista, Roraima, Brazil; ^2^ Department of Animal Biology, Federal University of Viçosa, Viçosa, Minas Gerais, Brazil; ^3^ Department of Fitopathology, Federal University of Viçosa, Viçosa, Minas Gerais, Brazil

**Keywords:** caterpillar, host specificity, leaf roller, *Salbia lotanalis*, weed

## Abstract

This work investigated eight species of Lepidoptera associated with *Miconia calvescens* DC. (Myrtales: Melastomataceae) in Brazil, including six defoliators, *Salbia lotanalis* Druce (Lepidoptera: Pyralidae), *Druentia inscita* Schaus (Mimallonidae), *Antiblemma leucocyma* Hampson (Noctuidae), three Limacodidae species, a fruit borer *Carposina cardinata* Meyrick (Carposinidae), and a damager of flowers *Pleuroprucha rudimentaria* Guenée (Geometridae). Based on host specificity and the damage caused to plants, *S. lotanalis* and *D. inscita* are the most promising species for biological control of *M. calvescens*. Furthermore, if *C*. *cardinata* and *P*. *rudimentaria* have host specificity in future tests, these caterpillars could also be considered as appropriate biocontrol agents.

## Introduction

Exotic plant species can become great threats to the biodiversity of natural environments and require expensive strategies for their control. In most cas control with herbicides and removal is expensive and inefficient. In some cases, integration of biological control and other strategies are also recommended. However, prior to the introduction, several steps are required, such as a survey of insects feeding on the plant in its region of origin, and an evaluation of damage and host specificity of selected candidate agents (Morin et al. 2009).

*Miconia calvescens* DC. (Myrtales: Melastomataceae) is a severe weed found in rainforest ecosystems on oceanic islands (Medeiros et al. 1997), and is classified among the one hundred worst invasive species around the world (Lowe et al. 2000; Murphy et al. 2008). *Miconia calvescens* is native to Central and South America and was introduced as an ornamental plant in French Polynesia, Hawaii, New Caledonia, and Australia (Meyer 1996; Csurhes 1997; Medeiros et al. 1997). On the Hawaiian and Tahitian Islands, the problems caused by the invasion of this weed are more serious, and many areas have become extensive monospecific stands of this plant (Meyer 1996; Medeiros et al. 1997). In Australia, the invasion of *M. calvescens* is more recent, but it has been classified as a major threat to the native biodiversity in the country (Murphy et al. 2008).

To select agents for the biological control of *M. calvescens*, surveys on the arthropods that attack this weed in Brazil have been carried out since 2001 (Picanço et al. 2005; Morais et al. 2010a, 2010b). In these previous studies, some species of Lepidoptera were found damaging *M. calvescens*, including *Salbia lotanalis* Druce (Lepidoptera: Pyralidae) (mistakenly identified as *Ategumia* sp.), *Druentia inscita* Schaus (Mimallonidae) and *Antiblemma leucocyma* Hampson (Noctuidae).

Several species of Lepidoptera have been extensively used in the biological control of invasive plants (Hight et al. 2002; Baars 2003; Ostermeyer and Grace 2007; Boughton and Pemberton 2009), and others have been considered as potential agents (Dhileepan et al. 2007). The major case of success was the introduction of *Cactoblastis cactorum* Bergroth (Lepidoptera: Pyralidae), a species native to Argentina that was introduced throughout the world for control of weedy *Opuntia* spp. (Caryophyllales: Cactaceae) (Pettey 1948). Among the main arthropod groups used in the biological control programs of weeds (Acarina, Coleoptera, Diptera, Hemiptera, Hymenoptera, Lepidoptera, and Thysanoptera), Lepidoptera were the fastest dispersers (Paynter and Bellgard 2011). The natural dispersal ability of a new agent is a limiting factor for success of biological control of weeds.

In fact, establishment and spread are two distinct issues in biological control programs of weeds. The population dynamics of an agent post—introduction can be influenced by factors such as climate (Dennill and Gordon 1990; Zalucki and van Klinken 2006), natural enemies (Hill and Hulley 1995; Paynter et al. 2010), resource availability (quantity and quality of host plant) (Foxcroft et al. 2007), and the appearance of more vigorous genotypes of weeds (Sheppard 1992; Nissen et al 1995). Crawley (1986) found that 44% of the failures of weed control by biological agents are to due the non-adaptation of these organisms to climate conditions in the country of introduction. Surveys of abundance of possible biocontrol agent can be used to improve understanding of the climate effects on the agent and of its establishment in new areas (Zalucki and van Klinken 2006). According to Paynter and Bellgard (2011), the knowledge about parameters of a biocontrol agent, prior to its introduction (agent voltinism, parasitoid diversity in the native range, habitat, fecundity, taxon and life—style category) could be used to predict if it will establish and disperse in a new environment.

This work presents the damage, host specificity, population dynamics, and the field occurrence of natural enemies of Lepidoptera species associated with *M. calvescens* in Brazil; the defoliators *S. lotanalis*, *D*. *inscita*, and *A. leucocyma*, three Limacodidae species, the fruit borer *Carposina cardinata* Meyrick (Carposinidae) and the flower damaging *Pleuroprucha* rudimentaria Guenée (Geometridae). The mass—rearing techniques of these species are also described.

## Materials and Methods

### Population dynamics

The population density of Lepidoptera associated with *M. calvescens* was evaluated in the municipalities of Viçosa, Dionísio, and Guaraciaba in Minas Gerais, Brazil. The experimental area in Viçosa was located in a secondary forest fragment under an advanced stage of natural recovery. The area was located at an altitude of 700 m (20° 46′ 37″ S, 42° 50′ 36″ W). During the experimental period, the average temperature was 19.4 ^°^C, and rainfall reached 1221 mm/yr.

In Dionísio, the experimental area was located in a riparian forest at the margins of the Rio Doce in an area belonging to a private company (CAF Santa Barbara Ltea.). The locality was at 344 m altitude and was located at 19° 50′ 34″ S, 42° 46′ 36″ W. During the experimental period, the average temperature was 23.2 °C, and rainfall reached 1003 mm/yr.

The experimental area in Guaraciaba was located in an abandoned pasture, under an initial natural recovery process, and was 1 km away from the Rio Piranga. This area was 580 m in altitude and located at 20° 34′ 36″ S, 43° 02′ 26″ W. During the evaluation period, the average temperature was 19.6° C, and rainfall reached 988.5 mm/yr. *Miconia calvescens* plants in this area were discovered at the beginning of 2005; therefore, evaluations at this site started only in that year.

Evaluations were performed during three different periods: the first period spanned June 2001 to June 2002; the second period was from February 2004 to February 2005; and the third period was from March 2005 to February 2006. During the first period, the plants were assessed every three weeks in Viçosa and Dionísio. During the second period, evaluations were carried out at 10-day intervals in Viçosa and monthly in Dionísio. In the third period, evaluations were made every two weeks in Viçosa and Guaraciaba.

Each evaluation involved the randomised sampling of ten *M. calvescens* plants, each one with a height of 1–7 m and a basal trunk diameter of 6–15 mm. For each plant, the number of Lepidoptera was counted in the main stem, six secondary branches, six inflorescences, and six infructescences. Leaves and buds from the secondary branches were examined. Six infrutescences were also brought to the laboratory to determinate whether Lepidoptera larvae were present inside the fruits. During the evaluations, the organ that each Lepidoptera attacked and the type and level of the damage caused by each species were recorded.

Representative samples of these species were deposited in the Museu Regional de Entomologia of the Universidade Federal de Viçosa.

Climate data from Viçosa were obtained from the climate station belonging to the Universidade Federal de Voçoa; data for Guaraciaba were obtained from the climate station at Ponte Nova (also belonging to the Universidade Federal de Viçosa); and data for Dionísio came from the Ponte Alta Climate Station (belonging to the private company CAF Santa Bárbara Ltda).

### Population dynamics: Hawaiian biotype

In September 2005, 10 *M. calvescens* seedlings of Hawaiian biotype were transplanted into the ground at the experimental area in Guaraciaba. These seedlings were used because plants from Hawaii have a distinct appearance, with leaves slightly softer and different in color (purple in abbatial face; in Brazilian plants this part is green). Therefore, the objective of this study was observed whether the seedlings of Hawaiian biotype were naturally attacked by caterpillars.

Seeds were collected in Hawaii and germinated in a box with sand. When the seedlings were at a height of 30—40 cm, they were transplanted into the ground at Guaraciaba the experimental area next to *M. calvescens* plants that grew naturally. Seedlings were arranged in a row with 2 m spacing between the plants. From September 2005 to February 2006, the natural attacks on these seedlings by caterpillars were also assessed during the site visits. Evaluations were made until February 2006 because after this time, the seedlings did not establish in the field due to defoliation by ants (*Atta* spp.).

### Effect of climatic parameters on caterpillar population dynamics

Canonical correlations and Pearson correlations were carried out to determinate the relationship between the population density of Lepidoptera and the climatic parameters (average, maximum, and minimum air temperature, relative humidity, rainfall, photoperiod, and wind speed). Statistical analyses were performed using the SAS procedures PROC CANCOR and PROC COR. In these analyses, the related to Lepidoptera density of each evaluation and the averages of climatic parameters 15 days preceding the evaluations were applied, except for rainfall, for which the total rain in the 15 days before the evaluations was applied.

### Parasitism

During the population peaks of Lepidoptera, 10 caterpillars of each species were collected in the field and brought to the laboratory to verify the emergence of parasitoids. These caterpillars were maintained in a rearing room with a light regime of 12:12 L:D, 26 ±2 °C air temperature, and 70 ±10% relative humidity. The defoliator caterpillars were placed in plastic bags (40 ×50 cm) with leaves of M *calvescens.* Fruit borer caterpillars were placed into transparent 2 L plastic pots (14 cm diameter, 14 cm height) with fruits inside. Flower feeding caterpillars were put inside wooden cage (0.5 ×1.0 ×1.2 m), enclosed in organza containing inflorescences of *M. calvescens.* The bags, pots, and cages that contained the caterpillars were maintained in a rearing room until the caterpillars pupated. The number of parasitized caterpillars and the number of emerged adult parasitoids of each Caterpillar were counted every day. The adult parasitoids were collected and forwarded to Dr. Maria Angélica Penteado-Dias for species identification.

### Host specificity: field conditions

During the evaluations, all vegetation in the 2 m radius of *M. calvescens* sampled during the population dynamics experiment was also observed to check whether the Lepidoptera species had attacked other plant species. Among the species found close to *M. calvescens*, other Melastomataceae were found. The most common were as follows: *Clidemia capitellata* (*Bonpl*.) D. Don., *Clidemia hirta* (L.) Don, *Leandra lacunosa Cogn, Miconia albicans* (Sw.) Triana, *M. mendoncaei* Cogn., *Miconia ibaguensis* (Bonpl.), and *Ossala confertiflora* (DC). Organs of these species were carried to the laboratory to determinate whether Lepidoptera larvae were present inside.

### Host specificity tests: *Salbia lotanalis*

*Host specificity tests of S. lotanalis were* carried out in glasshouses. These tests were conducted with the following Melastomataceae species: *C. capitellata, C. hirta, M. mendoncaei, M. albicans, M. ibaguensis, L. lacunosa, O. confertiflora, Tibouchina granulosa* (Desr.) Cogn., and *T. moricandiana* (DC.) Baill. The Hawaiian biotype of *M. calvescens* was also included in the tests.

The seeds of the Melastomataceae species were extracted from fresh fruits of trees located in the field where the evaluations were performed. The seeds of the Hawaiian biotype of *M. calvescens* were collected in Hawaii. The seeds were germinated in a box with sand. After about three months, when seedlings were 10–20 cm tall, they were transplanted to 5 L pots with soil. When the seedlings were 70–100 cm tall, four seedlings of each Melastomataceae species were put in a wooden cage (0.5 ×1.0 ×1.2 m), enclosed in organza and infested with five *S. lotanalis* caterpillars of the third instar. The caterpillars were reared in rooms similar to that described above. The instar of the caterpillars, their survival and the damage they caused to the seedlings were recorded daily.

### Mass—rearing

*Salbia lotanalis, D. inscita, A. leucocyma, C*. *cardinata,* and *P. rudimentaria were* reared in rearing rooms similar to the room described above. Caterpillars of *S. lotanalis, D. inscita,* and *A. leucocyma* were collected and placed into transparent plastic bags (40 ×50 cm) with leaves of *M. calvescens.* Five *M. calvescens* leaves were placed in each bag along with five caterpillars of similar size. These bags were maintained in a rearing room until the caterpillars pupated. The pupae were separated by sex according to characters described by Butt and Cantu (1962) and placed into plastic pots with wet vermiculite to avoid desiccation.

Emerged adults were transferred to a wooden cage (0.5 ×1.0 ×1.2 m) and enclosed in organza with a young *M. calvescens* plant (0.8–1.0 m high). 10 pairs were placed inside each cage and fed with a 10% honey solution. After the eggs hatched, the caterpillars were transferred with a fine brush to leaves of *M. calvescens*, which were put inside plastic bags (40 ×50 cm) and maintained in a rearing room, following the same procedures mentioned above.

*Miconia calvescens* fruits attacked by *C*. *cardinata* were collected in the field and put in into transparent 2 L plastic pots (14 cm diameter, 14 cm height). Each pot received about 80 g of fruit. The pots were capped with organza and their bottoms were covered with paper for absorbing the dampness of the fruits. When the fruits began to rot, the caterpillars were removed and transferred to pots containing fresh fruits. As caterpillars attained the pupa stage outside the fruits, the pupae could be seen and removed from the pots. The pupae were separated by sex according to characters described by Butt and Cantu (1962) and placed into plastic pots with wet vermiculite. The emerged adults were transferred to pots containing mature fruits of *M. calvescens*. 10 pairs of *C*. *cardinata* were put in each pot, and they were fed with a 10% honey solution. Emerged caterpillars from these pots were reared as mentioned above.

Caterpillars of *P. rudimentaria* were reared in wooden cages (0.5 ×0.5 ×0.5 m) and enclosed in organza with *M. calvescens* inflorescences. The inflorescences were collected in the field and maintained with their peduncle immersed in glass bottles containing 100 mL of water. 20 caterpillars of the same instar and four inflorescences were put in each cage. The inflorescences were changed every five days. Pupae were separated by sex according to characters described by Butt and Cantu (1962) and placed into plastic pots with wet vermiculite. The emerged adults were transferred to wooden cages (same characters described above) with four *M. calvescens* inflorescences. 10 pairs of *P. rudimentaria* were placed in each cage, and they were fed with a 10% honey solution. After the eggs hatched, caterpillars were transferred to new *M. calvescens* inflorescences and reared as mentioned above.

## Results

### Salbia lotanalis

**Figure 1.**
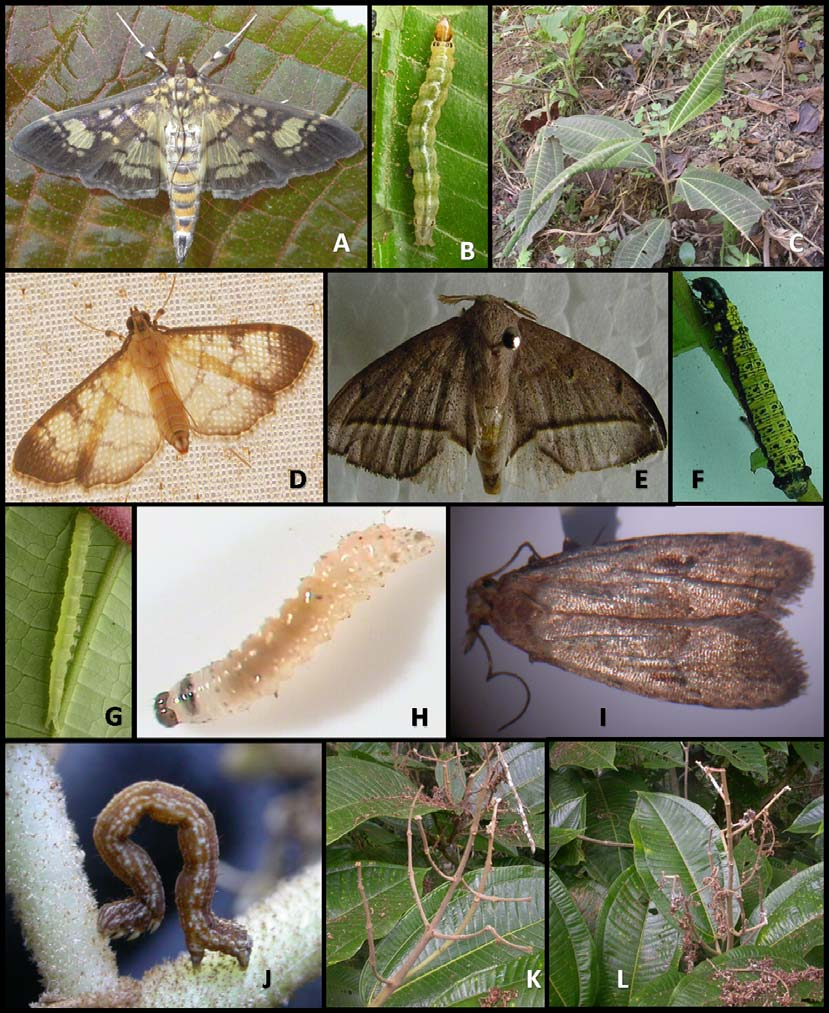
(A)Adult, (B) caterpillar, and (C) damage caused by *Salbia lotanalis* to *Miconia calvescens* seedlings (Hawaiian biotype); (D) adult of *Ategumia matutinalis;* (E) adult and (F) caterpillar of *Druentia inscita;* (G) caterpillar of *Antiblemma leucocyma;* (H) caterpillar and (I) adult of *Carposina cardinata;* (J) caterpillar and (K—L) damage caused by *Pleuroprucha rudimentaria* to inflorescences of *Miconia calvescens.* High quality figures are available online.

*Salbia lotanalis* is a leaf roller. Its caterpillars roll leaves longitudinally, forming tubes where they feed and eventually pupate. One caterpillar is able to destroy an entire leaf, causing its abscission. Adults are small brown moths with yellowish patterns on the wings and caterpillars are green with black patterns on the body (Figure 1A—C).

**Figure 2.**
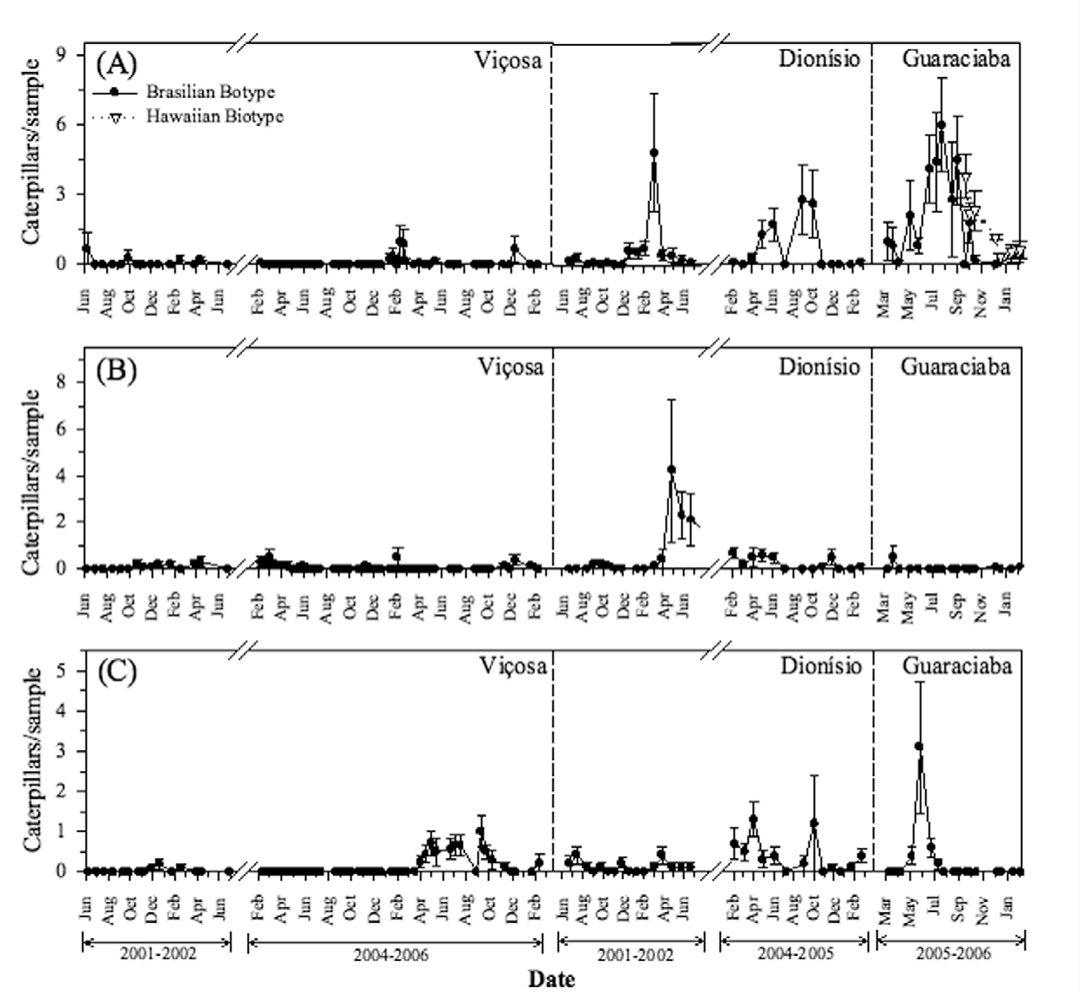
Population dynamics of leaf feeders: (A) *Salbia lotanalis* in plants of Brazilian and Hawaiian biotypes, (B) *Druentia inscita* and (C) *Antiblemma leucocyma* in Viçosa, Dionísio and Guaraciaba, Minas Gerais, Brazil, between 2001–2006. Vertical bars = standard error. High quality figures are available online.

The density of *S. lotanalis* was higher in Dionísio and Guaraciaba than Viçosa. In Dionísio, population peaks occurred from January to April 2002 and from May to October 2004. In Guaraciaba, *S. lotanalis* caterpillars were found throughout the whole evaluation period, with population peaks from June to September 2005. In Viçosa, *S. lotanalis* caterpillars were found occasionally and at a low density (Figure 2A). During population peaks, the damage caused by these caterpillars to *M. calvescens* plants was high, amounting to 50% defoliation. In these periods, some *M. calvescens* seedlings (Brazilian biotype) died due the high levels of defoliation by this leaf roller.

*Salbia lotanalis* and one species of Limacodidae were observed attacking sentinel plants (Hawaiian biotype) at Guaraciaba (Table 1). Two weeks after the sentinel plants were taken to the field *S.* *lotanalis* caterpillars were already found on these plants. The highest intensity of attack occurred from September to December 2006 (Figure 2A). In this period, all seedlings were attacked by at least one caterpillar. Furthermore, in December, one seedling died due to the damage caused by this caterpillar species. Only four Limacodidae caterpillars were found defoliating the seedlings in October.

**Table 1.**
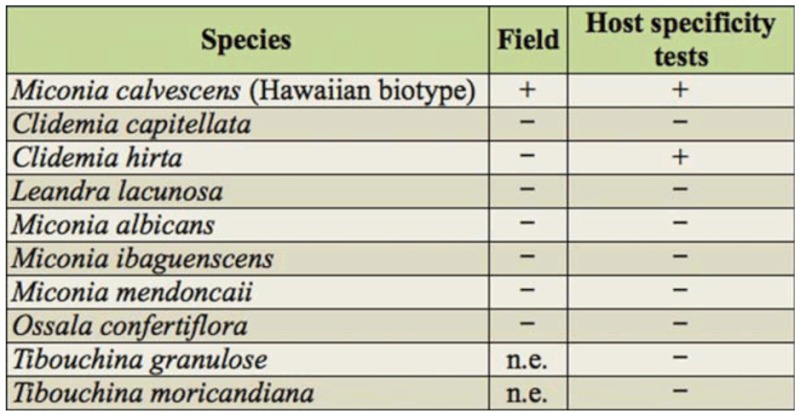
Host range of *Salbia lotanalis* as revealed by attack (+) or non—attack (-) on Melastomataceae species in field evaluations and in host *specificity tests.*

Nearly 10% *of S. lotanalis* caterpillars brought from the field were parasitized. Three parasitoid species were found: two Hymenoptera [*Apanteles* sp. (Braconidae) and *Pediobius* sp. (Eulophidae)] and a Diptera (Tachinidae). *Apanteles* sp. larvae emerged from the last instar of *S. lotanalis* and pupated outside the caterpillars. The pupae of this braconid are white, and about 20–30 larvae emerged from each caterpillar. Pupae of Tachinidae were also found in the last instar of *S. lotanalis*. The pupae of this Diptera are brown, and about 6–10 pupae emerged in each caterpillar. Three *S. lotanalis* pupae were also parasitized by Conura sp. (Hymenoptera: Chalcididae) in October 2004.

In the field, *S. lotanalis* caterpillars were not found on other plants species, including the other Melastomataceae assessed (Table 1). However, a similar species, *Ategumia matutinalis* Guenée, was observed attacking *C. hirta*. *Ategumia matutinalis* caterpillars are very similar to *S. lotanalis*, with similar wing color patterns as adults (Figure 1D). In host specificity tests, *S. lotanalis* caterpillars were able to develop through to pupation on both *C. hirta* and the Hawaiian biotype of *M. calvescens*. In other Melastomataceae, no damage by caterpillars was verified, and they died of starvation (Table 1).

### Druentia inscita

*Druentia inscita* caterpillars are also leaf rollers. They feed mostly on apical foliage, which they can completely destroy. The caterpillars are yellow with brown stripes and the adults are brown with two darker stripes on the wings (Figure 1E—F). Similarly to *S.* *lotanalis, D. inscita* caterpillars are found singly inside the leaf tubes. This species was also more abundant in Dionísio, with population peaks from May to June 2002 and from February to June 2004. In Viçosa, this caterpillar species was found occasionally and at low density. In Guaraciaba, it was found only in March 2005 (Figure 2B).

No parasitism was verified in *D. inscita*. No caterpillars of this lepidopteran were found in other plant species during the field evaluations, including the other Melastomataceae that were assessed (Table 1).

### Antiblemma leucocyma

*Antiblemma leucocyma* caterpillars are defoliators that make irregular holes in leaves. They are green and have a distinctive *looping walk* (*Figure 1E—F*), *and were found in all three places* (Figure 2C). Caterpillars of this species were found during the evaluation period in Dionísio. In Viçosa, their population peaks occurred from April 2005 to February 2006, while in Guaraciaba, they occurred only from May to July 2005 (Figure 2C).

About 30% of the *A*. *leucocyma* caterpillars brought from the field were parasitized by Hymenoptera (*Apanteles* sp. (Braconidae)). Many *A. leucocyma* caterpillars found in the field had dark coloration, indicating that they were parasitized. Caterpillars of this Lepidoptera were not found in the other plant species assessed (Table 1).

### Limacodidae

Three species of Limacodidae caterpillars were found occasionally in Viçosa, Dionísio, and Guaraciaba, causing defoliation of *M. calvescens* plants. These caterpillars were also found on the other sampled plant species neighboring *M. calvescens,* including plants belonging to the Melastomataceae family.

### Carposina cardinata

**Figure 3.**
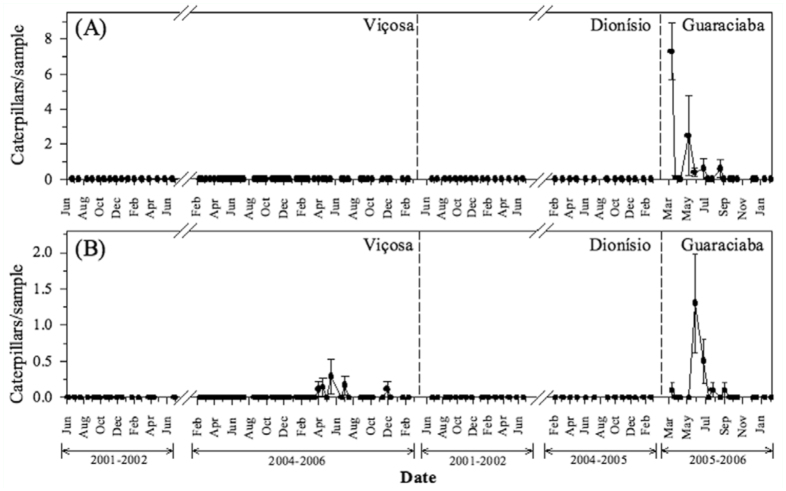
Population dynamics of reproductive organ feeders: (A) *Carposina cardinata* and and (B) *Pleuroprucha rudimentaria* in Viçosa, Dionísio and Guaraciaba, Minas Gerais, Brazil, between 2001–2006. Vertical bars = standard error. High quality figures are available online.

*Carposina cardinata* is a fruit borer of *M. calvescens* and is found only in Guaraciaba. Its caterpillars are beige and in the last instar they measure about 3 mm long. Adults are micro moths, bright gray, and measure about 5 mm (*Figure 1H*—*I*). They live inside fruits and feed on the flesh and seeds, causing severe damage. One to five caterpillars were found in each *M. calvescens* fruit. *Carposina cardinata* caterpillars were found in Guaraciaba in 2005, from March to September, which corresponded to the period of *M. calvescens* fruit production (Figure 3A). No parasitized caterpillars were found. During this period, the following Melastomataceae were also in their fruiting periods: *C. capitellata, C. hirta, L. lacunosa*, *M. albicans*, *M. mendoncaie*, *M. ibaguenscens*, and *O. confertiflora*. However, no *C*. *cardinata* was found in the fruits of any of these species (Table 1).

### Pleuroprucha rudimentaria

*Pleuroprucha rudimentaria* caterpillars feed on flowers. These caterpillars are brown, with white stripes and a distinctive *looping walk* (*Figure 1J*—*L*). This species was found only in Viçosa and Guaraciaba. In Viçosa, caterpillars attacked *M. calvescens* from April to June 2005 and in December 2005. In Guaraciaba, they were present from May to September 2005 (Figure 3B). During these population peaks, about 40% of inflorescences were completely stripped by this caterpillar species, and the fruit production of the *M*. *calvescens* plants was extremely low. No *P*. *rudimentaria* caterpillars brought to the laboratory were parasitized. This caterpillar was not found in other plant species, including the other Melastomataceae assessed (Table 1).

**Table 2.**
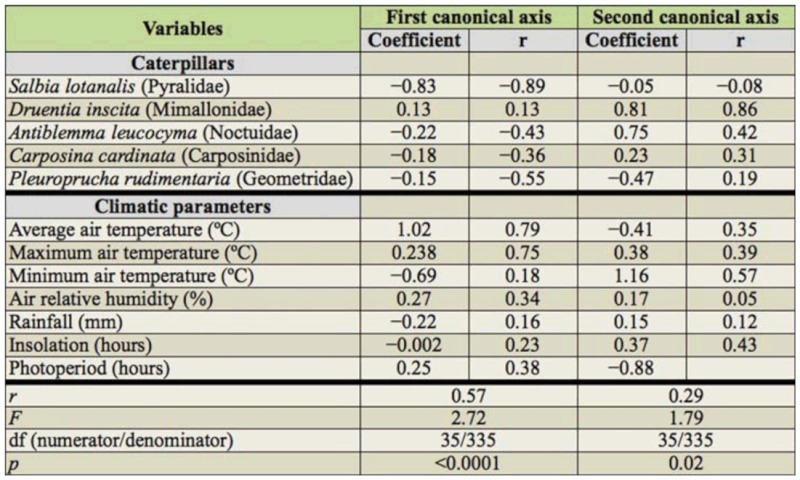
Canonical correlations and canonical pairs between the abundance of Lepidoptera associated with *Miconia calvescens* and climatic parameters in Viçosa, Dionísio and Guaraciaba, Minas Gerais, Brazil.

### Effect of climatic parameters on caterpillar population dynamics

There was a significant canonical correlation between the density of each Lepidopteran associated with *M. calvescens* and climatic parameters (Wilks' Lambda = 0.35, *F* = 2.72, df _num/den_= 35/335, *p* < 0.01). Five canonical axes were generated, out of which only the first two were significant at 5% probability and explained 86% of the correlation. According to the first canonical axis, among the Lepidoptera, *S. lotanalis* was the most influenced by the climatic parameters (r = -0.89), and according to the second axis, *D*. *inscita* (r = 0.86) was the most influenced by the climatic parameters. The climatic parameters that most affected the Lepidoptera density were the maximum (r = 0.75) and average (r = 0.79) air temperature according to the first axis, and the minimum air temperature (0.57) according to the second axis (Table 2).

**Figure 4.**
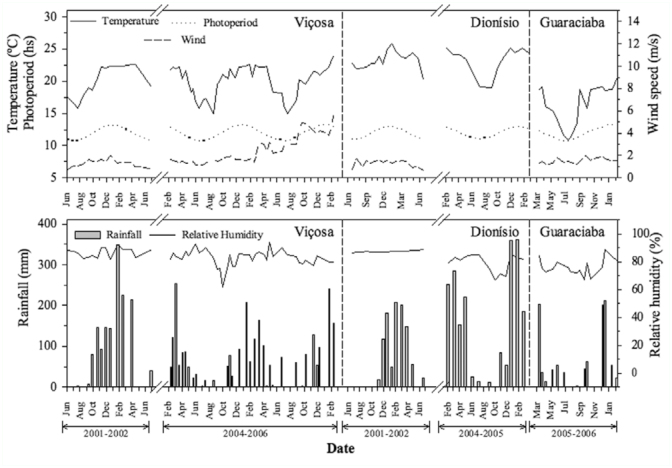
Average air temperature (°C), photoperiod (hours), wind speed (m/s), relative humidity (%), and total rainfall (mm) in Viçosa, Dionísio and Guaraciaba, Minas Gerais, Brazil, between 2001–2006. High quality figures are available online.

There was a positive Pearson correlation among the minimum, maximum, and average air temperatures and the population density of *D*. *inscita* (r = *034*,*p* < 0.01, r = *0.25*,*p <* 0.01 and r = 0.28, *p*< 0.01, respectively). In fact, highest densities of *D*. *inscita* were observed Dionísio, where air temperatures were higher (28.6, 19.6, 23.2 °C maximum, average, and minimum air temperatures, respectively) than Viçosa (27.2, 15.1, 20.1 °C maximum, average, and minimum air temperatures, respectively) and Guaraciaba (22.9, 16.3, 16.3 °C maximum, average, and minimum air temperatures, respectively) (Figure 4). There was a negative Pearson correlation among the maximum and average air temperatures and the population density of *S*. *lotanalis* (r = -0.38, *p* < 0.01 and r = -0.39, *p* < 0. 01, respectively). On the other hand, *S*. *lotanalis* was more frequent in Guaraciaba, where the temperatures were lowest. In addition the population peaks of this caterpillar occurred in colder months (Figure 4).

### Mass—rearing

*Salbia lotanalis, D*. *inscita,* and *A. leucocyma* can be reared throughout the year under laboratory conditions. As *M*. *calvescens* trees maintain a large number of leaves throughout the year, the caterpillars of these species have plenty of food throughout the year. Furthermore, *M*. *calvescens* leaves stayed in good condition for a week when they were kept in the plastic bags. *Salbia lotanalis* was the easiest to rear in the laboratory because of its shorter life cycle (about 70–100 days), high reproductive rate (∼90 eggs/female), and low mortality rate (more than 70% of individuals reached the adult stage). The life cycles of *D. inscita* and of *A. leucocyma* are longer, lasting 150–200 and 100–120 days, respectively. The reproductive rate of both these species is smaller (about 30 eggs/female).

*Carposina cardinata* and *P*. *rudimentaria* can be reared in the laboratory only during the flowering and fruiting periods of *M*. *calvescens*. Only two generations of these Lepidoptera were reared in the laboratory because afterwards there were no fruits or flowers in the field for feeding the caterpillars or for egg—laying by the adults. *Carposina cardinata* caterpillars survived and fed on fruits maintained in plastic pots. Their adults also laid eggs in *M*. *calvescens* fruits inside the pots. *Pleuroprucha rudimentaria* caterpillars fed on the inflorescences brought from the field, and adults laid their eggs in these inflorescences.

## Discussion

*Salbia lotanalis* has already been recognized as a potential biological control agent due its high reproductive capacity and impact on young plants (Morais et al. 2010b). The constant presence of *S. lotanalis* in the field under a range of climatic conditions indicates that this lepidopteran may establish well in new areas if introduced as a classical biocontrol agent against *M*. *calvescens*, since natural enemies that are frequent in the native range are likely to become frequent in the introduced environment (Sheppard 2002). The host specificity study demonstrated that *S*. *lotanalis* could feed on the leaves of *M*. *calvescens* and *C*. *hirta*. Additionally, its caterpillars were not found in other species in the field. Although this species may attack *C*. hirta in the new range, this does not hinder its introduction as a biocontrol agent for *M*. *calvescens*, because *C*. *hirta* is also a major invasive outside the neotropics (Wester and Wood 1977; Medeiros et al. 1997). In case *S*. *lotanalis* attacks *C. hirta* in the Pacific islands this would be regarded as highly beneficial.

A closely related leaf roller, *Salbia haemorrhoidalis* was used in successful programs for the biological control of *Lantana camara* in South Africa. This species showed good establishment and distribution in this country, as well as high population densities (Baars 2003). Another great advantage of employing *S*. *lotanalis* as a biocontrol agent is that it can be easily reared under laboratory conditions, with high population growth rates (Morais et al. 2010).

*Druentia inscita* also causes severe damage to *M*. *calvescens* plants in the field. During the evaluations, dead branches were observed due to the attack of this caterpillar on terminal buds. Nakahara et al. (1992) verified that *D*. *inscita* is restricted to 13 species of Melastomataceae out of the 39 species surveyed, and no larval feeding damage was observed on non—Melastomataceae from the 15 families tested. *Druentia inscita* was introduced in Hawaii for the control of *C*. *hirta*, occupying upper—elevation sites and shaded environments (Nakahara et al. 1992). However, its establishment was not very successful probably because few individuals were introduced (Culliney and Nagamine 2000). No records of it attacking *M*. *calvescens* were recorded after its introduction in Hawaii.

Compared with *S*. *lotanalis* and *D*. *inscita*, *A. leucocyma* caused less damage to *M*. *calvescens*, and there were long periods where this species was not found in the field. The low density of *A. leucocyma* may be due to the high parasitism rates by *Apanteles* sp. However, the indications of a high host specificity of this Lepidoptera is of relevance for its use as a biological control agent to be used against *M*. *calvescens* (Badenes-Perez and Johnson 2008). A similar caterpillar, *Antiblemma acclinalis* Hübner, from Trinidad was introduced into Hawaii for the control of *C*. *hirta* and had a confirmed establishment only in Oahu (Culliney and Nagamine 2000; Conant 2002).

*Carposina cardinata* is an important fruit borer of *M*. *calvescens*. Attacks of this lepidopteran can reduce seed fruit production. Studies have verified that herbivores of reproductive structures can be very effective weed biocontrol agents because they can reduce recruitment to the seedling stage of the populations (Bellows and Headrick 1999). A similar *Carposina*, *C. bullata* Meyrick, was used as a biocontrol agent of *C*. *hirta* in Hawaii, where it established and became very effective. This species feeds internally in the flower buds and is specific to the *Miconia* and *Clidemia* and the damage to the flowers of *C*. *hirta* prevents seed production (Nakahara et al. 1992). The major difficulty in the introduction of *C*. *bullata* was obtaining sufficient numbers of individuals to be released because no mass—rearing method was developed (Culliney and Nagamine 2000). Here, we verified that *C*. *cardinata* could be reared in the laboratory during the fruiting period of *M*. *calvescens* and a great number of adults could be obtained. Another example of *Carposina* used in control of weed is the seed—moth, *C*. *autologa* introduced in South Africa to control a serious invasive plant *Hakea sericea*, a small tree Australian origin (Gordon and Fourie 2011).

*Pleuroprucha rudimentaria* is also a potential biocontrol agent for *M*. *calvescens*. Fruit production decreased about 40% in the flowering period of 2005 in Viçosa and Dionísio possibly owing to this insect's attack. Furthermore, an *ad hoc* observation of a severe attack was observed in Viçosa in 2006 (Morais, personal observations). *Pleuroprucha rudimentaria* is reported to occur in Costa Rica, Peru, Jamaica, and Venezuela, but there is no information about its host specificity (BLDS 2010). During the field evaluations, no caterpillars of *P*. *rudimentaria* and *C*. *cardinata* were found attacking others plant species. Therefore, it would be worth conducting additional host— specificity tests for these species.

Parasitism may be a limiting factor for establishment of lepidopteran species in new areas. Native parasitoids have reduced the efficacy of some biocontrol agents, such as *Neogalea sunia* and *S*. *haemorrhoidalis* in the control of *Lantana camara* (Hill and Hulley 1995; Baars and Neser 1999). Nevertheless, although *S*. *lotanalis* has been parasitized by several species, this caterpillar maintains its high density in the field, causing severe damage to *M*. *calvescens*. Moreover, no parasitism was verified in *D*. *inscita*, *C*. *cardinata*, *or P. rudimentaria.*

Climate is also an important factor for the establishment of any biocontrol agent. Climatic factors can limit the dispersal of biological agents to new areas within the geographic range of the weeds (Baars 2003). Air temperature is one of the most important factors interfering with the establishment, dispersal, and impact of organisms in new areas of distribution (Baker 2002). *Salbia lotanalis* seems to develop better in periods of cooler air temperatures and *D*. *inscita* in hotter seasons (Figures 2A and 4).

The introduction of multiple herbivores as biocontrol agents against a single weed species may be advantageous, particularly where they attack different plant parts and suppress plant growth and reproduction. In this case, the introduction of *S*. *lotanalis* and *D*. *inscita* (defoliators) and *C*. *cardinata* and *P*. *rudimentaria* (attacking reproductive organs) might be a good strategy for the control of *M*. *calvescens*. Although *S*. *lotanalis* and *D*.*inscita* are defoliators, *S*. *lotanalis* feed on mature leaves, and *D*. *inscita* feed on young leaves in terminal buds, which prevents competition between them. Furthermore, *S*. *lotanalis* and *D*. *inscita* showed differences in their temporal distribution; *S*. *lotanalis* occurs in cooler periods, and *D*. *inscita* occurs in warmer periods. The same pattern was observed for *C*. *cardinata* and *P*. *rudimentaria*, the first species feeds on fruits and the second feeds on flowers. An effective result in the control of *Sesbania punicea* was obtained with the combined action of a seed—feeder *Rhyssomatus marginatus*, two defoliators *Tyria jacobaeae*, and a flea beetle *Longitarsus jacobaeae* (McEvoy et al. 1990; Hoffmann and Moran 1992).

Considering the difficulty and high cost of *M*. *calvescens* control by chemical or mechanical removal (Burnett et al. 2007; Hester et al. 2010), the introduction of effective biocontrol agents is regarded as necessary to reduce the population density of *M*. *calvescens* in Tahiti and Hawaii. Therefore, the traits of *S*. *lotanalis* and *D*. *inscita*, such as host specificity, high population growth rate, high rate of damage to the plants, relatively easy mass—rearing under laboratory conditions, and occurrence in the field throughout the year indicate that these lepidopterans can become good classical biocontrol agents against *M*. *calvescens*. Furthermore, if *C*. *cardinata* and *P*. *rudimentaria* have a sufficient degree of host specificity demonstrated in future tests, these caterpillars could also be considered for use as biocontrol agents.
